# Mixed method evaluation of Relational Team Development (RELATED) to improve team-based care for complex patients with mental illness in primary care

**DOI:** 10.1186/s12888-019-2294-1

**Published:** 2019-10-15

**Authors:** Danielle F. Loeb, Samantha Pelican Monson, Steven Lockhart, Cori Depue, Evette Ludman, Donald E. Nease, Ingrid A. Binswanger, Danielle M. Kline, Frank V. de Gruy, Dixie G. Good, Elizabeth A. Bayliss

**Affiliations:** 1 Division of General Internal Medicine, University of Colorado School of Medicine, Academic Office 1; Mailstop B180; 12631 East 17th Ave., Aurora, CO 80045 USA; 2 Lowry Family Health Center, Denver Health, Denver, CO USA; 3 Adult and Child Consortium for Health Outcomes Research and Delivery Science (ACCORDS), University of Colorado, Aurora, CO USA; 4 Kaiser Permanente Washington Health Research Institute, Seattle, WA USA; 5 Department of Family Medicine, University of Colorado, Aurora, USA; 6 Kaiser Permanente Colorado Institute for Health Research, Aurora, CO USA; 7 Department of Family Medicine, University of Colorado School of Medicine, Aurora, CO USA

**Keywords:** Multiple Chronic Conditions, Mental Disorders, Implementation Science

## Abstract

**Background:**

Patients with mental illness are frequently treated in primary care, where Primary Care Providers (PCPs) report feeling ill-equipped to manage their care. Team-based models of care improve outcomes for patients with mental illness, but multiple barriers limit adoption. Barriers include practical issues and psychosocial factors associated with the reorganization of care. Practice facilitation can improve implementation, but does not directly address the psychosocial factors or gaps in PCP skills in managing mental illness. To address these gaps, we developed Relational Team Development (RELATED).

**Methods:**

RELATED is an implementation strategy combining practice facilitation and psychology clinical supervision methodologies to improve implementation of team-based care. It includes PCP-level clinical coaching and a team-level practice change activity. We performed a preliminary assessment of RELATED with a convergent parallel mixed method study in 2 primary care clinics in an urban Federally Qualified Health Center in Southwest, USA, 2017-2018. Study participants included PCPs, clinic staff, and patient representatives. Clinic staff and patients were recruited for the practice change activity only. Primary outcomes were feasibility and acceptability. Feasibility was assessed as ease of recruitment and implementation. Acceptability was measured in surveys of PCPs and staff and focus groups. We conducted semi-structured focus groups with 3 participant groups in each clinic: PCPs; staff and patients; and leadership. Secondary outcomes were change in pre- post- intervention PCP self-efficacy in mental illness management and team-based care. We conducted qualitative observations to better understand clinic climate.

**Results:**

We recruited 18 PCPs, 17 staff members, and 3 patient representatives. We ended recruitment early due to over recruitment. Both clinics developed and implemented practice change activities. The mean acceptability score was 3.7 (SD=0.3) on a 4-point Likert scale. PCPs had a statistically significant increase in their mental illness management self-efficacy [change = 0.9, *p*-value= <.01]. Focus group comments were largely positive, with PCPs requesting additional coaching.

**Conclusions:**

RELATED was feasible and highly acceptable. It led to positive changes in PCP self-efficacy in Mental Illness Management. If confirmed as an effective implementation strategy, RELATED has the potential to significantly impact implementation of evidence-based interventions for patients with mental illness in primary care.

## Background

Mental illness complicates care for chronic medical illness. Patients with both mental and physical illness report lower quality of life and have higher medical costs, poorer outcomes, and higher mortality rates than those with only physical illness [1–15]. Approximately half of treatment for psychiatric conditions in the US occurs in the primary care setting [16–19]. Therefore, improving the diagnosis and treatment of mental illness in primary care is critical to improving outcomes among patients with chronic medical illness. Team-based models of care based on the chronic care model such as the Collaborative Care Model [20–24] have been shown to improve outcomes for complex patients, defined here as those with both chronic medical and mental illness. However, the uptake of these models by primary care clinics has been limited and wide gaps remain in the implementation of evidence-based models of care for patients with mental illness in primary care [25].

Primary care providers (PCPs) generally express high levels of satisfaction with team-based care for patients with mental illness [26–28]. Transitioning to team-based care can be challenging for primary care practice [26–40]. Practical and psychosocial aspects of practice change influence the processes of adopting and implementing team-based care. Practical factors include the need for additional training; changes to the work space requirements; workforce availability; revised workflows; billing and reimbursement challenges; and documentation procedures.

Key psychosocial aspects of implementing practice change involve clinic climate, relational coordination, and role clarity. Clinic climate is an organizational trait that involves the presence of a shared vision of the organization; psychological safety; concern for high quality; and support of innovation [41]. Relational coordination, a theory of organizational management focusing on the interdependent relationships between people working in teams, predicts the quality of care in healthcare settings. Three relational domains — shared goals, shared knowledge, and mutual respect — overlap aspects of clinic climate but focus more directly on the interpersonal relationship between team members [42, 43]. Clinic climate and relational coordination among members of clinical teams have been shown to predict the quality of chronic disease care, and poor clinic climate can represent a significant barrier to successful practice facilitation [41, 44–49]. In implementing team-based care, clinic staff and PCPs from diverse professional backgrounds need to communicate and collaborate successfully [43, 50, 51]. However, many physicians, nurses and clinic staff are not trained in the communication skills required for high-level relational coordination. For PCPs, moving to a team-based model represents a significant change in their role in the clinical team, which can be confusing and threatening to professional self identity. PCPs can view their role within the healthcare team differently from how other team members, such as social workers and nurse practitioners, view physicians’ roles within the team, which can lead to tensions [31, 37]. Thus, role clarity (mutual understanding and respect for the expertise among members of the care team) is critical in implementation of team-based care.

Additionally, PCPs have low confidence in their knowledge and skill when caring for patients with mental illness. In previous qualitative and quantitative studies, they have expressed the need for more support in caring for complex patients with mental illness [52–56]. Thus, PCP’s may need focused training in the transition to a team-based approach and in the management of mental illness within a team-based approach.

A commonly used implementation strategy for team-based care is practice facilitation. Practice facilitation utilizes practice facilitators to assist clinics and healthcare systems in implementing practice change [46, 57, 58]. Practice facilitation is effective at improving communication across specialties [57, 59, 60], increasing adoption of practice change [61, 62], and building consensus [63]. Practice facilitators often have specialized training in mental illness and have been utilized in training providers in the management of mental illness [64, 65]. However, while standard practice facilitation can support implementation of team-based care models by addressing practical aspects of change, it does not explicitly address many of the psychosocial factors essential to sustainable practice change. Improved implementation strategies for team-based care should also address team climate, relational coordination, and clinician self-efficacy.

To address psychosocal factors of implementation, we developed Relational Team Development (RELATED), a brief clinic-wide intervention that combines team-level practice facilitation and PCP-level clinical coaching [66]. RELATED is an enhanced practice facilitation strategy that draws upon psychological clinical supervision models for the PCP-level clinical coaching. Psychotherapy clinical supervision has been shown to lead to increased knowledge of and increased acquisition of psychotherapy skills among both psychotherapists and psychiatric nurses [67, 68]. Further, RELATED utilizes practice facilitators with a background in clinical psychotherapy, as they have specialized training in interpersonal dynamics, communication, and conflict resolution. Table 1 illustrates the distinguishing features between RELATED and standard practice facilitiation.

**Table 1:** Components of Relational Team Development (RELATED) and Standard Practice Facilitation

	RELATED		Standard Practice Facilitation
	PCP Clinical Supervision and Coaching (Coaching)	Practice Change Activity Team (PCAT)	
Description	Facilitator observes PCPs in 4+ visits with complex patients; facilitator uses clinical psychology and coaching techniques during 1-on-1 debriefs with PCPs.	**Facilitator guides implementation of a practice change**; in this process, maladaptive team dynamics are identified and addressed.	**Facilitator guides implement-ation of a practice change** and builds internal capacity for improvement activities.
Participants	• PCPs • Patients whose visits are observed	• **Clinic team (i.e., PCPs and staff representatives, leadership)** • Patient representatives	• **Clinic team (i.e., PCP and staff representatives, leadership)**
Implementation Factors	**Distinguishing Features**		
Mental Illness Management ⇒ Knowledge ⇒ Skills ⇒ Communciation	• Diagnostic and treatment feedback • Didactics tailored to individual knowledge gaps • Patient communication practice • Multicultural case discussion	• Tailored group didactics on mental illness • Communication practice	
Practical ⇒ Quality improvement processes ⇒ Practice monitoring systems ⇒ Improvement Plans ⇒ Modified Workflows	• Use of interdisciplinary team and available mental health resources	**Training:** • **Quality improvement methods** • **Measurement-Based Care (MBC)** **Facilitation:** • **Assess current system** • **Create improvement plan (for MBC)** • **Implement improvement plan (for MBC)** • **Evaluate/modify improvement plan targeting sustainability**	
Psyschosocial ⇒ Interpersonal relationships ⇒ Clinic Culture ⇒ Attitudes ⇒ Role change ⇒ Role clarity	Interpersonal focus on: • Emotional reactions • Self and other awareness • Attitudes towards team-based care • Experience of clinic culture	Team dynamics focus on: • Non-hierarchical communication and leadership behaviors • Creating mutually agreed upon processes • **Role clarity** • Psychological safety • Past practice change experiences	Group process emphasizing: • **Role clarity** • Communication workflows • Team-building activites

Bolded text indicated shared features

In this study we evaluated the feasibility and acceptability and conducted a preliminary assessment of efficacy of the RELATED intervention to address implementation barriers for team-based models of care. Although RELATED can be used to implement more general practice change, this study evaluated RELATED specifically in the implementation of team-based care for complex patients who had chronic medical and mental illness.

## Methods

### Study design

We utilized a convergent parallel mixed method approach to evaluate the acceptability and feasibility as well as preliminary efficacy of the RELATED intervention. The initial iterative development of the RELATED intervention is previously described [66]. We continued the iterative development of RELATED by conducting consecutive pilot studies in two clinics and making modifications to the intervention between the first and second clinic pilot.

### Intervention

In RELATED, a practice facilitator with graduate training in psychotherapy utilizes a subset of psychotherapy clinical supervisory activities and standard practice facilitation techniques in a two-part intervention. RELATED includes 1) Clinical Supervision and Coaching (Coaching) in which a facilitator shadows PCPs during visits with patients who have complex needs and provides feedback on clinical care and utilization of team-based care; and 2) Practice Change Activity Team (PCAT) in which the facilitator guides the PCP participants, their clinical teams, and patient representatives as they work through an evidence-based practice change activity. Patient representatives are included in the PCAT to provide insight from the patient perspective of potential changes to clinic processes. Table 1 outlines in detail the intervention components. Team-based care and mental health didactics are delivered to PCPs, staff, leadership and patient representatives during the PCAT component. Didactics are drawn from a comprehensive educational resource including mental health and team-based care competencies that were developed by the practice facilitator and principal investigator (D.F.L). Mental health competencies include mood disorders and anxiety disorders. Team-based care competencies include components of the Chronic Care Model, Complex Leadership Theory, and quality improvement process approaches. Complex leadership theory acknowledges the complex nature of healthcare systems in which leadership emerges from the dynamic relationships between members of the system that foster the adaptive capacity of healthcare systems. Complex adaptive systems allow for the self-organized emergence of outcomes that may surpass those of a hierarchical, mechanical system [69, 70]. The practice facilitator determines which didactics are most appropriate for the group based on group dynamics and subject matter interest. Human subjects review: The study was approved by the Colorado Multiple Institutional Review Board.

### Setting

We pilot tested RELATED in two primary care clinics in urban Federally Qualified Health Centers that are part of a safety net integrated healthcare system in the Southwest, USA between 2017 and 2018. Both clinics serve racially and ethnically diverse patient populations. We utilized a sequential approach to performing the intervention with time to refine RELATED processes in the period between the two clinics. The first clinic included internal medicine, family medicine, and medicine-pediatrics primary care specialties. The second clinic only included internal medicine. Both clinics had integrated psychologists and social workers on the care team providing mental illness consultation to PCPs, short-term psychotherapy to patients, and connection to the local specialty mental health system. One clinic had two full-time behavioral health providers and the other had two part-time behavioral health providers. Both clinics utilized the Primary Care Behavioral Health model of integration, offering patients access to mental health care can often be inaccessible to patients [71].

### Participants and Recruitment

Participants included clinician PCPs, practice staff and patient representatives. Additionally, patients gave written consent to have their PCP visits shadowed during the Coaching component. Currently practicing PCPs (MDs/DOs, Nurse Practitioners, and Physician Assistants) were recruited for the full intervention. PCPs were recruited in a face-to-face meeting during their regularly scheduled provider meeting supplemented by follow up emails. Resident physicians were excluded from the study due to schedule limitations. For the Coaching component, patient visits were identified by the practice facilitator and approved for shadowing by the PCP. Patients were approached prior to their visit by the practice facilitator for informed consent to have their visit shadowed. Patients were eligible if they met our definition of complexity—patients with at least one mood or anxiety disorder and one chronic medical illness. Patients were excluded if they were unable to consent due to language other than Spanish and English or cognitive delay. Clinic staff and leadership were recruited for the PCAT. Eligible clinic staff members included nurses, medical assistants, social workers, behavioral health providers, patient navigators, addiction counselors, clinic leadership, and front desk staff. The intervention was explained by the study team in a regularly scheduled all-clinic meeting, and the clinic leadership decided which staff would be invited to participate in the intervention. In addition, the practice facilitator, who treats patients in a different primary care clinic within the healthcare system, helped identify and contact eligible patient representatives using a phone script to see if they were interested in participating in the PCAT portion of the intervention. Patients were contacted by the practice facilitator up to three times. All participants signed written consent.

## Evaluation

We used a convergent parallel mixed method approach to the evaluation [72, 73]. Our primary outcomes were feasibility and acceptability. Secondarily we assessed preliminary efficacy of the RELATED intervention. We used quantitative and qualitative methods as complementary evaluation means to gain a more complete assessment of RELATED (Additional file 1 for reporting standards). We used quantitative surveys to assess intervention acceptability and to evaluate changes in PCP measures pre- and post- intervention (Additional file 2 for PCP survey). Qualitative focus groups helped provide a more nuanced perspective on the acceptability and feasibility of the intervention. Finally, field notes collected by the research team helped assess clinic climate to complement the survey and focus group results.

### Data collection

#### Primary outcomes

Primary outcomes were acceptability and feasibility of RELATED. Acceptability was measure through an acceptability survey and focus groups. Feasibility was measured in ease of recruitment and implementation of RELATED.

#### Secondary outcomes

We surveyed participating PCPs pre- and post-intervention to assess change in self-efficacy in mental illness management and team-based care; communication self-efficacy; knowledge of mental illness management; attitudes toward team-based care; and team climate.

Further, field notes from observations were used to document daily clinic routines and workflows, document interactions among staff and between staff and PCPs, and to gain a better understanding of the overall clinic climate.

##### Survey instruments

We adapted the Healthcare Provider Acceptability of a Behavioral Intervention to Promote Adherence to apply to RELATED [74]. It consisted of 4 items and is scored on a 4 point Likert scale from “completely disagree” to “completely agree”. Questions addressed included: time involved in intervention, setting for intervention, appropriateness of intervention, and impact of intervention.

The PCP pre- and post-intervention survey consisted of several validated measures. We previously developed and validated measures of PCP self-efficacy in mental illness management and team-based care [75, 76]. The mental illness management (10 items) and team-based (7 items) scales are scored on a 0 to 10 Likert scale where 0 is “not at all confident” and 10 is “extremely confident”. We modified validated instruments to measure changes in communication self-efficacy, knowledge of mental illness management, attitude toward team-based care and team climate [77–81]. We chose a limited number of items from each instrument using a combination of published factor loadings and content domains. Communication was assessed with a modified version of the Communication Skills Self-assessment, which is focused on provider-patient communication [77]. The four-point response scale was modified to the 11-point scale used for the team-based care and mental illness management self-efficacy scales. The self-assessment was reduced from 35 questions to 11 questions. To assess provider attitudes, we selected five items from the 26 item Attitudes toward Health Teams Scale [78]. Likewise, to assess team climate we used six items of 61 from the Team Climate Inventory which focused on clinical team function and communication. Higher scores indicated more positive attitudes toward team-based care and team climates. We constructed summary variables for the following domains: Communication Skills Self-Assessment, Attitudes Toward Health Teams Scale and Team Climate Inventory by calculating the mean score for each set of items within the scale. Mental health treatment knowledge was assessed with a version of Katerndahl’s instrument that assesses mental health treatment for major depressive disorder (MDD), generalized anxiety disorder (GAD), and panic disorder adapted to include bipolar disorder. Participants were asked to rate how effective (not effective, somewhat effective, effective, or don’t know) each of 14 different psychotropic medications were for MMD, GAD, and bipolar disorder. Higher scores indicated higher mental illness treatment knowledge [81]. These modifications were made and initially utilized in a state-wide survey of Colorado PCPs. These methods were previously described [75, 76].

##### Survey procedures

Surveys to assess acceptability were administered on paper to participating PCPs and clinical staff at 1 and 6 months after the start of the intervention. The PCP survey was administered in person prior to the start of the intervention and after the intervention. PCP surveys took approximately 10 minutes to complete. All surveys were de-identified.

##### Focus groups

We conducted three focus groups at each site. Staff and patients were included in the same focus group because the patients were integrated into the PCAT along with staff. Separate focus groups were conducted with PCPs and clinic leadership to encourage the members of each group to feel safe in expressing themselves. The focus groups were semi-structured to allow for a comprehensive and systematic, yet flexible approach [82]. We assessed acceptability and feasibility of the intervention; the effects of the intervention on team dynamics; sustainability of the intervention; and suggested changes to the intervention. The focus groups were 60 minutes each; they were conducted by research assistants; lunch was served as an incentive. The focus groups were audio recorded and transcribed. In focus groups in the first clinic, we elicited feedback on suggested changes to improve RELATED.

##### Clinic climate

In addition to focus groups, we utilized a modified ethnographic approach to better understand clinic climate [83]. We used less intrusive methods for primary data collection and limited methods that would require provider or staff time away from patients. Data collection included field notes by the practice facilitator and site coordinator. The same research assistants who help conduct the focus groups also shadowed staff in workflow observation. In each clinic at least one clerk, medical assistant, nurse, behavioral health specialist, and social worker were shadowed for a half-day.

##### Analysis

Quantitative data were analyzed using SAS version 9.4 software. All statistical tests were performed with a level of significance of 0.05. Self-efficacy subscales, as well as attitudes toward team-based care, team climate, and acceptability scales were scored by calculating mean of responses across all items in the patient care domain for each provider. Knowledge of effective treatments of mental illness subscales were scored as the percent of correct answers in each domain for each time period. Pre-post evaluations of the patient care domains were performed with paired t tests.

For both the focus groups and clinic observations, we used an iterative and team-based process guided by qualitative content analysis, and data were collected until saturation of themes was reached [84, 85]. Two qualitatively trained analysts both inductively and deductively developed a code book. Initial domains for codes were based on the interview guide domains and the codebook was expanded based on codes that emerged from the data. The analysts jointly reviewed and coded the dataset of focus group transcripts and observation field notes until no new codes were identified and there was strong code assignment agreement. The practice facilitator provided member checking throughout code book development. All transcripts were independently read, double coded, and then merged prior to analysis. Any discrepancies in coding were addressed through discussion and consensus among the coders. Throughout the analytic process, the analytic team (qualitative analysts) met regularly with the broader study team to check new findings, discuss emergent new codes and themes, and assess the preliminary and final results. ATLAS.ti version 8.0 was used for data organization and management.

## Results

We recruited 18 PCPs for the full intervention in the two clinics. We only sent one recruitment email to PCPs at each clinic, as we met or exceeded our goal of eight per clinic following this initial invitation. PCPs were predominantly female, middle age and non-Hispanic White. We recruited 12 physician and six non-physician PCPs. Fifty-eight patients agreed to have their visits shadowed for the coaching component. For the PCAT only, we recruited 20 clinic staff (including non-PCP leadership) and 3 patient representatives in the two clinics. The PCPs recruited for the full intervention also participated. Staff were primarily female and White and about a third Hispanic ethnicity. About half of the patients whose visits were shadowed in the Coaching component identified as Hispanic. Many patients indicated race as “other” or did not indicate (Table 2). All PCPs and staff completed the study. One patient representative in the PCAT was withdrawn due to concern raised by their PCP about PCAT participation. PCAT attendance averaged 63% and 67% among PCPs and staff, respectively, in clinic one and 75% and 65% for PCPs and staff, respectively, in clinic two. One patient completed 5 of 6 of the PCAT sessions and completed the focus group. One patient completed 3 of 6 of the PCAT sessions and did not participate in the focus group.

**Table 2:** Primary Care Provider, Patient, and Staff Demographics

	Providers (*N*=18)	Patients (*N*=58)^a^	Staff (*N*=20)
Clinics N (%)			
Clinic 1	10 (56)	34 (59)	9 (45)
Clinic 2	8 (44)	24 (41)	11 (55)
Gender N (%)			
Female	12 (67)	43 (74)	18 (90)
Male	6 (33)	14 (24)	2 (10)
Unknown/Missing	0	1 (2)	0
Age M (SD)	39 (7)	45 (14)	36 (10)
Race N (%)			
African-American/Black	1 (6)	3 (5)	0
American Indian/Alaskan Native	1 (6)	4 (7)	0
Asian (includes Southeast Asian, Indian)	3 (17)	0	2 (10)
Pacific Islander/Native Hawaiian	0	1 (2)	1 (5)
Caucasian/White	11 (61)	28 (48)	12 (60)
Multiple races	0	1 (2)	0
Other	1 (6)	8 (14)	4 (20)
Unknown/Missing	1 (6)	13 (22)	1 (5)
Ethnicity N (%)			
Hispanic	2 (11)	26 (45)	8 (40)
Non-Hispanic	16 (89)	27 (46)	12 (60)
Unknown/Missing	0	5 (9)	0
Professional Background N (%)			
Nurse Practitioner	4 (22)		
Physician	12 (67)		
Physician Assistant	2 (11)		
Medical Specialty N (%)			
Family Medicine	7 (39)		
Medicine-Pediatrics	1 (6)		
Internal Medicine	10 (56)		
Years since completing residency N (%)			
Missing	3 (17)		
10 – 19	4 (22)		
5-9	4 (22)		
< 5	7 (39)		
Diagnoses M (SD)			
Number of Total Medical Diagnoses		4.7 (3.9)	
Number of Mental Illness		1.5 (0.7)	

*N* Number, *SD* Standard Deviation, *M* Mean

^a^Patients in the PCAT were recruited from those shadowed in the Coaching component. Two were recruited in Clinic 1 and one in clinic 2. One patient from Clinic 1 participated in focus group. No patients from Clinic 2 completed PCAT or focus group

Staff and PCP participants found the intervention highly acceptable. Mean scores on the acceptability measure at one month were 3.8 (Standard Deviation = 0.3) on a 1-4 Likert scale with N=36. Mean scores at six months after the start of the intervention were 3.7 (SD = 0.4) with N= 33. All 18 PCPs completed the pre- and post- intervention surveys. Although not designed to power for changes in the pre-post surveys, there was a statistically significant improvement in PCP mental illness management self-efficacy and in knowledge in treating major depressive disorder (Table 3). Scores are reported as combined between two clinics. There was no significant difference between the two clinics.

**Table 3:** Pre-post Changes in PCP Survey Scores

Survey Scale/Subscale	Pre-Post Mean Difference (95%CI)	Paired T test *P*-value
Team Based Care SE (0-10)	0.8 (-0.3,1.9)	0.14
Mental Health Care SE (0-10)	**0.9 (0.5,1.4)**	**<.01** ^a^
Communication SE (0-10)	0.4 (-0.1,0.9)	0.09
Overall Knowledge of Treatment (0-100)	4.0 (-0.8,8.8)	0.10
Knowledge of MDD Treatment	**6.7 (0.1,13.3)**	**0.05** ^a^
Knowledge of GAD Treatment	2.9 (-4.3,10.2)	0.40
Knowledge of BPD Treatment	3.1 (-4.7,10.9)	0.42
Attitude Toward Team Based Care (1–5)	-0.1 (-0.3,0.1)	0.38
Team Climate (1-%	-0.1 (-0.4,0.3)	0.61

*N* = 18

*SE* Self-efficacy, *MDD* Major Depressive Disorder, *GAD* Generalized Anxiety Disorder, *BPD* Bipolar Disorder, ^a^statistically significant change

Bold-face are statistically significant by *p*-value

Focus group results complemented and reinforced the findings from the survey concerning acceptability and feasibility. In focus groups, the majority of participants described RELATED as valuable, efficient, and rewarding. PCPs overwhelmingly found the coaching sessions helpful and suggested adding more sessions. One PCP described feeling burdened by the time spent in the PCAT sessions. Leadership and staff had positive comments about the PCAT and, notably, did not express concerns about the time commitment. Staff and PCPs described positive effects of RELATED on team dynamics, inclusivity and patient-centeredness. It was also noted that RELATED supported a patient-centered approach by bringing the patient perspective to the forefront of practice change. Finally, RELATED was found to help level the traditional medical hierarchy, opening opportunities for staff previously not engaged in leadership behaviors to adopt them. See Table 4 for a summary of focus group results.

**Table 4:** Focus Groups Results

Acceptability and Feasibility	
Domain	Representative Quotes
Acceptability positive	I thought it went really well overall. I thought it was a great project. I really appreciated having you all come in. I thought it was a nice way to get the whole staff involved doing our project and learn a little bit about QI and really work as a team in an efficient manner...-PCP
	I think it’s been great things going here, the experience … I think it’s a good thing. I would think it’s a good thing for us to have this. It helped the clinic. -Staff
Feasibility positive	I liked how [practice facilitator] worked through the project because I think it was a little difficult in the beginning and helping us decide what we wanted to work, but I think [practice facilitator] did a really good job at narrowing it down and getting it to something that was attainable. –Staff Leadership
	[practice facilitator] was able to accomplish a lot in a sufficient amount of time where we weren’t like going into the next session or anything so…I thought [practice facilitator] managed it really well. -Staff
Feasibility negative	In terms of how many hours have we spent doing that [PCAT]. Even though in the world of QI it’s pretty efficient, for me it’s not. It’s probably ten hours in the past couple months… That’s a lot of time.”–PCP
Impact on Team	
Team functioning	It’s a different level of respect because now we have more of an understanding of what each of our role is, and how important it is once the patient reaches that certain person because we didn’t have an understanding of what their job entails, and how much work they’re putting in to it. –Staff
Hierarchy	It was nice to see other people speak up and take more of a leadership role in this. Our patient navigator and some other people who are really involved and passionate and to see the skills that those individuals had. It allowed them to go above their role and take on more. It was a good environment to hopefully put people on an equal playing field. –PCP
Patient perspective	It’s changed my perspective… It makes me a little bit more patient-centered when I deal with things… aware of what’s really going on in the clinic or why people are responding the way they are. –Staff
Inclusivity	I think it was good that it came from a variety of people so that no one made the decision all by themselves... It was interesting having all those groups together and being able to come together, make a decision and then be able to do it… I think it helped everyone so I think everyone got a little something from the changes –Leadership

In Clinic One focus groups, we also elicited suggested changes from PCPs and staff to inform the second pilot intervention in Clinic Two. PCPs and staff suggested several changes to the intervention. PCPs recommended changes to maximize their coaching time with the practice facilitator, such as scheduling complex patients when the practice facilitator was present; more shadowing sessions with the practice facilitator; and blocking off the last hour of their patient schedule for feedback from the practice facilitator. PCPs suggested changes to the feedback session, including more constructive feedback; educational materials offered during the sessions; and provision of information on best practices in communication. Regarding the PCAT component, PCPs suggested blocking off patient care time for the meetings and using graphics to track the practice change activity. They requested additional topics for the didactics, including opioid dependency, bipolar disorder, substance use disorders, personality disorders, and managing medications. Both PCPs and staff suggested inviting more staff to participate in the PCAT; creating ongoing follow-up on the practice change to check in on progress, separating the didactics from the PCAT and making the clinical didactics optional for staff. Clinic staff also suggested involving clinic leadership from the beginning.

Research staff shadowed 17 staff members from clinic one and eight from clinic two. Team interactions in PCAT sessions were also observed. Observations at the two pilot clinics highlighted key features of clinic climate that could impact practice change. Clinic one typically acted as a single unit; PCPs and staff displayed a “we” approach to caring for patients and there was cohesion among staff and providers/leadership. Clinic staff and PCPs displayed high levels of trust with each other. Clinic two appeared to function within the traditional medical hierarchy and emphasized individual performance. Both clinics demonstrated mostly positive work relationships and a significant amount of support among staff.

We made several adaptations to RELATED prior to clinic two based on feedback from clinic one: 1) The scheduling of the Coaching component was changed to mirror the consultation model used by the behavioral health providers in the clinics; the practice facilitator was available to Coach the PCPs from one clinical pod per clinic session rather than a single PCP per session. 2) Coaching feedback sessions were shortened to 5-7 minutes after each patient observation; 3) Brief didactic interventions were incorporated into the Coaching component; 4) A booklet was created for participants to track PCAT progress; 5) The time between the 5^th^ and 6^th^ PCAT sessions was increased to address any issues that arose in initial implementation of the practice change; 6) Clinic leadership was invited to join the PCAT from the beginning; 7) Additional staff were invited to join the PCAT; and 8) The mental health didactics were changed to mandatory for PCPs but optional for staff and patient representatives. After these changes were made and piloted in clinic two, no further suggestions were made by clinic two participants to change the intervention.

## Discussion

RELATED is a novel approach to implementing team-based approaches to care for mental illness in primary care settings. In this pilot, we demonstrate that RELATED is both feasible and highly acceptable among PCPs, clinic staff, and clinic leadership. Both quantitative and qualitative assessments of acceptability were positive. In fact, PCPs requested additional Coaching sessions. Focus group data was consistent across staff, PCPs and leadership, citing overall improvement in team functioning, including inclusivity across roles and a better understanding of the interdependence of others’ roles. We experienced minimal barriers to study recruitment or logistics of the intervention. Our primary feasibility challenge was incomplete attendance for the PCAT sessions. Although not designed to be powered for the PCP survey outcomes, PCPs had a significant increase in their mental illness management self-efficacy and improvement in their knowledge of treatment of major depression in pre- and post-intervention surveys. Although features of the clinic climate differed in the two pilot clinics, we found no significant differences in acceptability or feasibility results. We also found no difference in PCP survey results across clinics. We did not find a change in Attitudes Toward Team-based Care or Clinic Climate measures.

RELATED represents a promising new method for implementing practice change in the primary care setting, specifically for the care of patients with mental illness and chronic medical disease. Although RELATED builds on standard practice facilitation, it has key innovations. RELATED has a unique emphasis on improving PCP engagement in the care of patients with mental illness and with the team-based approach to care employed in their clinic. It increases engagement by improving PCP self-efficacy in the management of complex patients in the context of the healthcare team. This increased engagement is theorized to lead to practice change with more depth and sustainability than traditional practice facilitation. PCPs have shared discomfort with treating mental illness due to a lack of knowedge and training. They also have expressed poor self-efficacy in communicating with patients with mental illness [52, 55, 56]. By addressing these deficits, PCPs are given the opportunity to more effectively engage in the interdisciplinary care of their most complex patients.

In addition to the practical aspects of practice change, RELATED addresses both PCP-level and clinic-wide interpersonal relationships. RELATED utilizes a practice facilitator with specialized training in health psychology as well as traditional practice facilitation techniques. This background facilitates the clinical supervision of PCPs during the Coaching component of RELATED and the psycho-therapeutic training provides practice facilitators with enhanced skills to manage relational coordination dynamics during the PCAT. Although clinical supervision has been shown to increase knowledge of and acquisition of psychotherapy skills in diverse professional roles [67, 68], such supervision is not included in standard practice facilitation. Standard practice facilitation often involves coaching a practice team through an evidence-based quality improvement project. The PCAT is innovative in that it serves multiple purposes in RELATED. It is a vehicle for teaching the team the basic skills of practice change. Additionally, it serves as a lab for the team to improve their Relational Coordination skills and for PCPs to improve their self-efficacy in team-based care. These changes are intended to decrease barriers to care and practice change activities moving forward.

Despite the different features of clinic climate observed at each clinic, we were both surprised and encouraged that acceptability and PCP survey results did not differ between the two clinics. RELATED works directly with clinic climate in its focus on PCP communication skills and clinic interpersonal relationships. Although additional research is needed, this early finding may point to RELATED’s success in working with diverse clinic climates in the implementation of practice change. However, since we did not find changes in the Attitudes Toward Team-based Care or Clinic Climate measures, the effect of RELATED on these domains needs further evaluation. Both the positive and null findings need further confirmation.

The importance of clinic climate on practice change has been illustrated in studies of the patient centered medical home (PCMH). In a literature review of factors affecting implementation of intensive outpatient care in Veterans’ Affairs medical homes, Breland, et.al. found that factors in the inner clinical setting were key to success of the interventions including a culture of innovation, good systems for communication, clinic leadership engagement, and positive tension for change (i.e., the belief that there was a need for the change). They also noted characteristics of staff and leadership in the successful clinics, which included creativity, flexibility and strong interpersonal skills. Clinicians noted the importance of “a cohesive team of very skilled individuals” [86]. These findings are supported by a study of key facilitators and barriers to the implementation of PCMH in nine Minnesota practices, where clinic climate was seen as a key factor. The alignment of values with the practice change and openness to innovation were facilitators, while clinics with resistance to change and providers who felt they did not need the change were noted as barriers to implementation [87]. By targeting clinic climate, RELATED may significantly improve the implementation of practice change in primary care. RELATED’s focus on PCP-specific role clarity, clinic-wide interpersonal relationships, and clinic climate could all be applied to the implementation of a variety of practice change initiatives for chronic disease in general.

As a pilot, this study has multiple limitations. Our primary outcomes were acceptability and feasibility. Though we did evaluate effectiveness in PCP self-efficacy, communication, knowledge, attitudes and team-climate, we were not powered for those measures. We also did not evaluate RELATED for its effectiveness in implementing a specific practice change. As a next step, we plan to evaluate its effectiveness in implementing measurement-based care for anxiety and depression in primary care. The study was performed in clinics from one urban federally qualified, safety net integrated delivery system in the Southwestern United States. Therefore, the results are not necessarily generalizable to other types of primary care practices or other regions. We plan to conduct further trials in diverse clinic settings to test the generalizability of these findings. The intervention would need to be adapted to utilize external mental health resources for clinics without integrated behavioral health. Further, with testing in only two clinics, our findings on the effect of clinic climate, feasibility, acceptability, and PCP survey results can only be seen as generative. It will be important to further explore the ability of RELATED to impact implementation of team-based care with larger studies. In order to scale-up RELATED, we envision healthcare systems and clinics using the tools acquired in an intervention for the implementation of future practice change activities. Further research on the sustainability of the effects of RELATED would be necessary to test this hypothesis. RELATED is more resource intensive than traditional practice facilitation. Cost effectiveness will need to be evaluated prior to taking it to scale. This will include factors suggesting the additional cost of RELATED over traditional practice facilitation is warranted (e.g., clinics with a history of failed practice change efforts). Additionally, the use of practice facilitators with graduate training in psychotherapy is somewhat restrictive. However, given the increasingly widespread implementation of integrated behavior health in primary care, a workforce intimately familiar with the nuances of mental health care delivery in primary care is already in place that could be trained to deliver RELATED. We also plan a formal evaluation of the effect of RELATED on clinic Relational Coordination. The evaluation was performed by the research team, who were known to participants. Thus, these results may be subject to social desirability bias. Lastly, we had no control group to evaluate change in the PCP measures. In the pre- post design, PCPs are only controlled against their initial scores. This design is subject to selection bias, as only PCPs who volunteered to participate were evaluated.

## Conclusion

We successfully piloted the RELATED implementation strategy. RELATED was feasible and highly acceptable. It led to positive changes in PCP self-efficacy in mental illness management and team based care. Next steps involve conducting a larger trial to test the effectiveness of RELATED in implementing a specific evidence-based practice change to improve the clinical care of patients with mental illness in primary care. If confirmed to be effective as a strategy, the potential applications include training practice facilitators to work healthcare systems where they could assist individual practices with multiple practice change activities within a continuous practice improvement model. Individual practice facilitators could also be trained to work with multiple different small practices, and returning for follow-up practice change activities as needed. As a novel implementation strategy, RELATED has the potential to advance the field of practice change for patients with mental illness in primary care.

## Supplementary information

**Additional file 1:** Standards for Reporting Implementation Studies: the StaRI checklist for completion. Reporting standards for implementation.

**Additional file 2:** PCP survey. Pre- and Post- PCP survey.

## Data Availability

The datasets used and analyzed during the current study are available from the corresponding author on reasonable request.

## Abbreviations

PCPs: Primary Care Providers
RELATED: Relational Team Development
PCAT: Practice Change Activity Team
PCMH: Patient Centered Medical Home
