# Supplementary material for: Mixed method evaluation of Relational Team Development (RELATED) to improve team-based care for complex patients with mental illness in primary care
Source: BMC Psychiatry. 2019 Oct 15;19:299. doi: 10.1186/s12888-019-2294-1 (PMC6792180; doi:10.1186/s12888-019-2294-1)
Supplement: Supplementary file 2 — Additional file 2: PCP survey. Pre- and Post- PCP survey. [file 12888_2019_2294_MOESM2_ESM.pdf]

Division of General Internal Medicine
SCHOOL OF MEDICINE
UNIVERSITY OF COLORADO **ANSCHUTZ MEDICAL CAMPUS**

Guidelines:

- You are not required to answer every question in the survey.
- There is no right or wrong answer.
- Please answer based on your current practice and understanding, unless otherwise indicated.
- All of your answers will be kept strictly confidential and will be reported only in summaries; i.e., with the responses of other participants.
- For each question, select one answer either by circling the number associated with your answer or marking the appropriate box.

1) Please answer the following on a 0 – 10 scale regarding your confidence in the following domains of patient care. Circle 1 number for each question.

How confident are you that you can…	0 (not at all confident) – 10 (extremely confident)										
**diagnose** hypertension?	0	1	2	3	4	5	6	7	8	9	10
**treat** hypertension?	0	1	2	3	4	5	6	7	8	9	10
**diagnose** chronic obstructive pulmonary disease?	0	1	2	3	4	5	6	7	8	9	10
**treat** chronic obstructive pulmonary disease?	0	1	2	3	4	5	6	7	8	9	10
**treat** two or more co-morbid chronic medical illnesses?	0	1	2	3	4	5	6	7	8	9	10
**diagnose** major depression?	0	1	2	3	4	5	6	7	8	9	10
**treat** major depression?	0	1	2	3	4	5	6	7	8	9	10
**diagnose** generalized anxiety disorder?	0	1	2	3	4	5	6	7	8	9	10
**treat** generalized anxiety disorder?	0	1	2	3	4	5	6	7	8	9	10
**diagnose** bipolar disorder?	0	1	2	3	4	5	6	7	8	9	10
**treat** bipolar disorder?	0	1	2	3	4	5	6	7	8	9	10
**manage an acutely suicidal patient?**	0	1	2	3	4	5	6	7	8	9	10
**treat** your patients who have b**oth chronic medical** and **mental illness?**	0	1	2	3	4	5	6	7	8	9	10
**have a productive conversation** with a pulmonologist to care for a patient with chronic obstructive pulmonary disease?	0	1	2	3	4	5	6	7	8	9	10
**have a productive conversation** with a psychologist to care for a patient with bipolar disorder?	0	1	2	3	4	5	6	7	8	9	10
**have a productive conversation** with a psychiatrist to care for a patient with bipolar disorder?	0	1	2	3	4	5	6	7	8	9	10
**maintain ongoing trusting relationships** with the patients in your panel?	0	1	2	3	4	5	6	7	8	9	10
explain treatment options to your patient in a manner that ensures a high level of understanding by your patient?	0	1	2	3	4	5	6	7	8	9	10
help your patient cope with her or his worries by explaining the current medical problem to her/him in a manner that facilitates coping?	0	1	2	3	4	5	6	7	8	9	10
explain the possible benefits and risks to your patient of the recommended tests, procedures, and treatment options (including medications)?	0	1	2	3	4	5	6	7	8	9	10
convey empathy to your patient regarding her/his problem?	0	1	2	3	4	5	6	7	8	9	10

**How confident are you that you can…**	**0 (not at all confident) – 10 (extremely confident)**										
identify and pursue verbal cues given by your patient?	0	1	2	3	4	5	6	7	8	9	10
identify and pursue non-verbal cues given by your patient?	0	1	2	3	4	5	6	7	8	9	10
communicate effectively with your patient even though you find her/him to be rather difficult?	0	1	2	3	4	5	6	7	8	9	10
actively involve your patient in the process of making treatment-related decisions?	0	1	2	3	4	5	6	7	8	9	10
secure your patient's commitment to try to follow the treatment plan that you developed with your patient?	0	1	2	3	4	5	6	7	8	9	10
use the last few minutes of the encounter to summarize the important issues discussed during the encounter?	0	1	2	3	4	5	6	7	8	9	10

2) For the next 7 questions, refer to the following clinical scenario:

Please answer these questions even in you do not work in a team setting. Imagine you work in a primary clinic with a TEAM-BASED APPROACH to care. There are 3 clinical teams in the clinic. Your team includes: 1 nurse, 1 care manager, 5 medical assistants, 1 nurse practitioner, and 4 physicians.

The *care manager* is a nurse whose primary responsibility is to use databases to track the clinical care of patients in your team with chronic medical or mental illnesses.

The *care manager* focuses on the following chronic conditions: depression, diabetes mellitus, and hypertension. The *care manager* uses evidence-based treatment protocols to make treatment adjustments for these conditions. The *care manager* consults with you, the primary care provider, on patients who do not respond to treatment as expected.

Working as a physician in this clinical setting, how confident are you that you would be able to…

	**0 (not at all confident) – 10 (extremely confident)**										
**rely on the care manager** to work with your patients to **develop specific plans to meet their goals** for their chronic medical or mental illness(es)?	0	1	2	3	4	5	6	7	8	9	10
**rely on the care manager** to contact your patients to **monitor their progress toward meeting their goals** for their chronic medical or mental illness(es)?	0	1	2	3	4	5	6	7	8	9	10
**rely on the care manager** to contact patients in your panel with **diabetes** who are overdue for their **HbA1c to schedule them to come in to clinic for follow-up testing**?	0	1	2	3	4	5	6	7	8	9	10
**collaborate with the care manager who uses treatment algorithms to make medication adjustments** to improve blood pressure control in your patients with **uncontrolled hypertension**?	0	1	2	3	4	5	6	7	8	9	10
**collaborate with the care manager who uses treatment algorithms to make medication adjustments** to improve symptom control in your patients with **uncontrolled major depression**?	0	1	2	3	4	5	6	7	8	9	10
**encourage team members to contribute to creative solutions to improve** patient care within your practice?	0	1	2	3	4	5	6	7	8	9	10
**establish a positive atmosphere among the members of the clinical team?**	0	1	2	3	4	5	6	7	8	9	10

3a) How effective are the following medications at monotherapy (sole treatment) for Major Depression? Please do not consider side effects when answering this question.

	Not Effective	Somewhat Effective	Effective	Don't know
Amitriptyline (Elavil)	❑	❑	❑	❑
Trazodone	❑	❑	❑	❑
Lithium carbonate (Lithium)	❑	❑	❑	❑
Venlafaxine (Effexor)	❑	❑	❑	❑
Bupropion (Wellbutrin)	❑	❑	❑	❑
Haloperidol (Haldol)	❑	❑	❑	❑
Propranolol (Inderal)	❑	❑	❑	❑
Quetiapine (Seroquel)	❑	❑	❑	❑
Buspirone (Buspar)	❑	❑	❑	❑
Mirtazapine (Remeron)	❑	❑	❑	❑
Valproic acid (Depakote)	❑	❑	❑	❑
Clonazepam (Klonopin)	❑	❑	❑	❑
Paroxetine (Paxil)	❑	❑	❑	❑
Cognitive Behavioral Therapy	❑	❑	❑	❑

3b) How effective are the following medications at monotherapy (sole treatment) for Generalized Anxiety Disorder? Please do not consider side effects when answering this question.

	Not Effective	Somewhat Effective	Effective	Don't know
Amitriptyline (Elavil)	❑	❑	❑	❑
Trazodone	❑	❑	❑	❑
Lithium carbonate (Lithium)	❑	❑	❑	❑
Venlafaxine (Effexor)	❑	❑	❑	❑
Bupropion (Wellbutrin)	❑	❑	❑	❑
Haloperidol (Haldol)	❑	❑	❑	❑
Propranolol (Inderal)	❑	❑	❑	❑
Quetiapine (Seroquel)	❑	❑	❑	❑
Buspirone (Buspar)	❑	❑	❑	❑
Mirtazapine (Remeron)	❑	❑	❑	❑
Valproic acid (Depakote)	❑	❑	❑	❑
Clonazepam (Klonopin)	❑	❑	❑	❑
Paroxetine (Paxil)	❑	❑	❑	❑
Cognitive Behavioral Therapy	❑	❑	❑	❑

3c) How effective are the following medications at monotherapy (sole treatment) for Bipolar Disorder? Please do not consider side effects when answering this question.

	Not Effective	Somewhat Effective	Effective	Don't know
Amitriptyline (Elavil)	❑	❑	❑	❑
Trazodone	❑	❑	❑	❑
Lithium carbonate (Lithium)	❑	❑	❑	❑
Venlafaxine (Effexor)	❑	❑	❑	❑
Bupropion (Wellbutrin)	❑	❑	❑	❑
Haloperidol (Haldol)	❑	❑	❑	❑
Propranolol (Inderal)	❑	❑	❑	❑
Quetiapine (Seroquel)	❑	❑	❑	❑
Buspirone (Buspar)	❑	❑	❑	❑
Mirtazapine (Remeron)	❑	❑	❑	❑
Valproic acid (Depakote)	❑	❑	❑	❑
Clonazepam (Klonopin)	❑	❑	❑	❑
Paroxetine (Paxil)	❑	❑	❑	❑
Cognitive Behavioral Therapy	❑	❑	❑	❑

4) The following questions ask your opinion about working with a team of providers to care for your patient. Please indicate your level of agreement with each of the following statements by circling the number that best reflects your level of agreement for each statement.

**STATEMENT: "IN MY OPINION"**	Strongly Disagree	Disagree	Neutral	Agree	Strongly Agree
Developing an interdisciplinary care plan is excessively time consuming.	1	2	3	4	5
Working on a team keeps most health professionals enthusiastic and interested in their jobs.	1	2	3	4	5
In most instances, the time required for team meetings could be better spent in other ways.	1	2	3	4	5
The team approach permits health professionals to meet the needs of family caregivers as well as patients.	1	2	3	4	5
A team's primary purpose is to assist physicians in achieving treatment goals for the patient.	1	2	3	4	5
Physicians have the right to alter patient care plans developed by the team.	1	2	3	4	5

5) Please indicate your level of agreement with each of the following statements. Circle 1 option. Please answer the following questions in the context of your primary clinical practice site. The following questions ask about your "team". For these questions, please think of your "team" as the health care providers, nurses, and other staff at your practice or clinic.

**Statement**	**Strongly Disagree**	**Disagree**	**Neither Agree nor Disagree**	**Agree**	**Strongly Agree**
We have a "we are in it together" attitude.	1	2	3	4	5
People keep each other informed about work related issues in the team.	1	2	3	4	5
People feel understood and accepted by each other.	1	2	3	4	5
There are real attempts to share information throughout the team.	1	2	3	4	5
There is a lot of give and take.	1	2	3	4	5
We keep in touch with each other as a team.	1	2	3	4	5

6) What is your primary professional role?
(Check 1 option)

Clinician	☐
Clinician - Educator	☐
Clinician - Researcher	☐
Clinician - Administrator	☐

7) What is your medical specialty?
(Check 1 option)

Family Medicine	❑
Internal Medicine	❑

8) How many years has it been since you completed residency?
(Check 1 option)

< 5	❑
5 - 9	❑
10 - 19	❑
≥ 20	❑

9) What is your gender?
(Check 1 option)

Male	❑
Female	❑

9) What is your race?
(Check 1 option)

African-American / Black	❑
Caucasian / White	❑
Asian (includes Southeast Asian, Indian)	❑
Pacific Islander / Native Hawaiian	❑
American Indian / Alaskan Native	❑
Multiple Races	❑
Other	❑

9) What is your ethnicity?
(Check 1 option)

Hispanic	❑
Non-Hispanic	❑
